# The Methanesulfonamide Group: Bright and Dark Sides of hERG Potassium Channel Inhibition

**DOI:** 10.3390/ph19060882

**Published:** 2026-06-01

**Authors:** Zsigmond Máté Kovács, József Óvári, János Magyar, Tamás Bányász, Péter P. Nánási, Balázs Horváth, Gábor Balogh, Norbert Szentandrássy

**Affiliations:** 1 Department of Physiology, Faculty of Medicine, University of Debrecen, H-4032 Debrecen, Hungary; kovacs.zsigmond@med.unideb.hu (Z.M.K.); horvath.balazs@med.unideb.hu (B.H.); 2 Doctoral School of Dental Sciences, University of Debrecen, H-4032 Debrecen, Hungary; 3 Division of Sport Physiology, Department of Physiology, Faculty of Medicine, University of Debrecen, H-4032 Debrecen, Hungary; 4 Department of Dental Physiology and Pharmacology, Faculty of Dentistry, University of Debrecen, H-4032 Debrecen, Hungary; 5 Division of Clinical Laboratory Science, Department of Laboratory Medicine, Faculty of Medicine, University of Debrecen, H-4032 Debrecen, Hungary; 6 Department of Basic Medical Sciences, Faculty of Dentistry, University of Debrecen, H-4032 Debrecen, Hungary

**Keywords:** methanesulfonamide, hERG channel, I_Kr_, class III antiarrhythmics, action potentials

## Abstract

Our review focuses on methanesulfonamide-containing compounds, a well-characterized class of high-affinity blockers of the hERG potassium channel, which plays a critical role in cardiac repolarization by mediating the cardiac I_Kr_. These compounds, which include notable class III antiarrhythmic drugs such as dofetilide and d-sotalol, block the hERG channel in its open state by binding within the inner vestibule. This interaction is particularly strong with some residues and the compounds form hydrogen bonds with others. This binding results in high-affinity inhibition with slow dissociation kinetics, frequently leading to drug trapping and prolonged action potential duration (APD). This can predispose patients to arrhythmias, including Torsades de Pointes. Beyond cardiac drugs, there are several non-cardiac methanesulfonamide drugs that also block the hERG channel. This causes pro-arrhythmic side effects despite their primary indications. The clinical significance of these effects, especially in patients with impaired drug elimination, is that accumulation increases the risk of arrhythmia. The objective of forthcoming research endeavors is to mitigate hERG affinity, with the aim of reducing pro-arrhythmic risks while maintaining therapeutic efficacy. This means structural modifications that seek to remove or modify the methanesulfonamide group. Machine learning also emerged as promising tool for exploring drug–protein interactions. It is evident that the methanesulfonamide moiety plays a pivotal role in the structural basis of hERG blockade. However, it should be noted that this moiety does not necessarily represent a universal pharmacophore. This observation underscores the necessity for a nuanced approach in drug development, aimed at achieving a balance between efficacy and safety.

## 1. Introduction

In light of the demographic shift towards an ageing population, the necessity of medication is becoming increasingly pervasive. In the Northern American region, it has been documented that approximately 90% of individuals over the age of 65 receive prescription medication for one or more chronic illnesses [1]. Cardiac diseases are arguably the most significant of these. The heart is the organ responsible for blood circulation, ensuring that oxygen and nutrients reach all cells. Declining cardiovascular health can lead to subpar function in all other organs, which not only reduces human lifespan (cardiovascular diseases are the second most common cause of death in Europe, while it is the first in the United States [2]) but also has a significant impact on quality of life.

In the context of the mass production of synthetic drugs, companies have been observed to utilize the same molecule motifs or functional groups to achieve the same anti-bacterial, anti-viral, anti-neoplastic, etc., effects as their peers [3,4,5]. It is unfortunate that a significant proportion of our own cells possess structures capable of interacting with these drug molecules, a phenomenon that is especially prevalent in cases of overdose or compromised elimination, such as in instances of renal or liver failure. This interaction can result in the accumulation of the drug in the body in its unaltered form [6,7,8]. In consequence of contemporary drug development, there has been an enhancement in the specificity of the pharmaceuticals in question, whilst the probability of overdose has been reduced. Therefore, the occurrence of drug side effects is reduced; however, the incidence is not negligible.

It is imperative to undertake a comprehensive examination of drug molecules at the level of their target organ/cell, complemented by rigorous cross-examination with other critical but off-target organs/cells. Such a meticulous approach is crucial to ensure that the potential benefits of a drug outweigh its potential adverse effects. This is particularly important in the context of fragile neurons and muscle cells and fibers, which are vital for the proper functioning of the body.

### 1.1. What Is Methanesulfonamide and Its Applications in Drug Synthesis

Sulfonamides, including methanesulfonamides, are employed in the synthesis of various organic molecules, including pharmaceutical drugs. Sulfonamide-containing drugs were among the earliest synthetic antibiotics, with Prontosil being introduced in 1932 [9]. It has been demonstrated that Prontosil exerts its effect through the disruption of bacterial tetrahydrofolate formation [10]. It is evident that sulfonamides and their chemical alternatives, including methanesulfonamides, have been identified as effective in the treatment of non-bacterial diseases. This has consequently led to the development of numerous pharmaceutical agents that incorporate the pharmacophore, including diuretics, antivirals, antitumor and antiarrhythmic medications [10]. Sulfonamides represent a broad class of compounds characterized by the SO_2_-NH moiety. In contrast, methanesulfonamides constitute a structurally more defined subclass, wherein the sulfonyl group is substituted by a methyl group. The consequence of this is a reduction in steric bulk and an increase in conformational flexibility. Antiviral activity may be manifested through the inhibition of polymerase [11,12,13] or reverse transcriptase [14]. It has been demonstrated that they are capable of inhibiting COX-2 activity in a variety of tumors [15,16] when employed as anti-neoplastic agents. Finally, they have the capacity to trap themselves within ion channels, principally potassium channels, as a consequence of their comparatively large pore size and positive charge. The ensuing discourse will elucidate the mechanisms of action and the attendant consequences of this novel approach.

### 1.2. Molecular Details of hERG Blocking by Molecules Containing Methanesulfonamide Group

It is imperative to avoid unintentional drug binding to hERG during the development of new pharmaceuticals. To this end, computational techniques have been employed to facilitate the understanding of the binding of drug molecules to the channel well before the publication of the first experimentally determined structures. A significant source of information regarding the structural basis of binding was provided by mutagenesis studies, in which multiple mutations of hERG were examined. These mutations were found to result in the loss of interaction with the blockers. Amino acids that were found to contribute to the binding of multiple molecules include T623, S624, V625, G648, Y652, F656 and V659 [17,18]. Kalyaanamoorthy and Barakat have published a comprehensive review of the various computational prediction methods, providing a concise overview of several pharmacophore models for predicting drug interactions with hERG [19]. A number of studies utilized docking and homology models based structures of other receptors [20,21], whereas others employed three dimensional quantitative structure–activity relationship (QSAR) [22,23] or pharmacophore modelling [24,25] methodologies. In a number of these models, the position of a positively charged nitrogen atom (frequently located in a ring) and its relative position from aromatic rings or hydrophobic groups are critical for binding to the channel [22,23,25]. In contrast, other models involved molecules devoid of protonated nitrogen [20,21]. However, a divergence in the molecular details of binding has been observed between models, as evidenced in the extant literature [19]. It is evident that no pharmacophore model has been identified in which a specific role for the methanesulfonamide group has been hypothesized. Consequently, it is challenging to draw conclusions from these studies on the binding mechanisms of such molecules.

The cryo-EM structure for the open state of the channel, as published by Wang et al. in 2017 [26], corresponded to an open conformation of the channel. This structure has led to the development of enhanced computational models for understanding the basis of hERG blockade by drugs, including molecules containing a methanesulfonamide group. In a study by Emigh Cortez et al. [27], the binding of seven hERG ligands was investigated using molecular docking with RosettaDock to multiple models of the channel. These included the open and closed states (the latter being a model based on EAG1) as well as several known mutated variants known to affect ligand binding. In the course of the study, it was found that three of the seven molecules under investigation contained a methanesulfonamide group. These were dofetilide and the two enantiomers of sotalol. The docking results indicate that the sulfonamide group of dofetilide interacts with residues T623 and S624 via hydrogen bonds. These results indicate that these residues also contributed to the interaction of sotalol with the channel along with Y652. The authors have identified a significant disparity in the binding of these three molecules to the channel in comparison to the remaining four: a reduced propensity to bind into the hydrophobic pocket. This finding is at odds with an earlier detailed docking study involving 233 molecules, in which a homologous model was used for the channel [28]. In this instance, the methanesulfonamide group of dofetilide exhibited significant interaction with I655 and F656.

**Figure 1:**
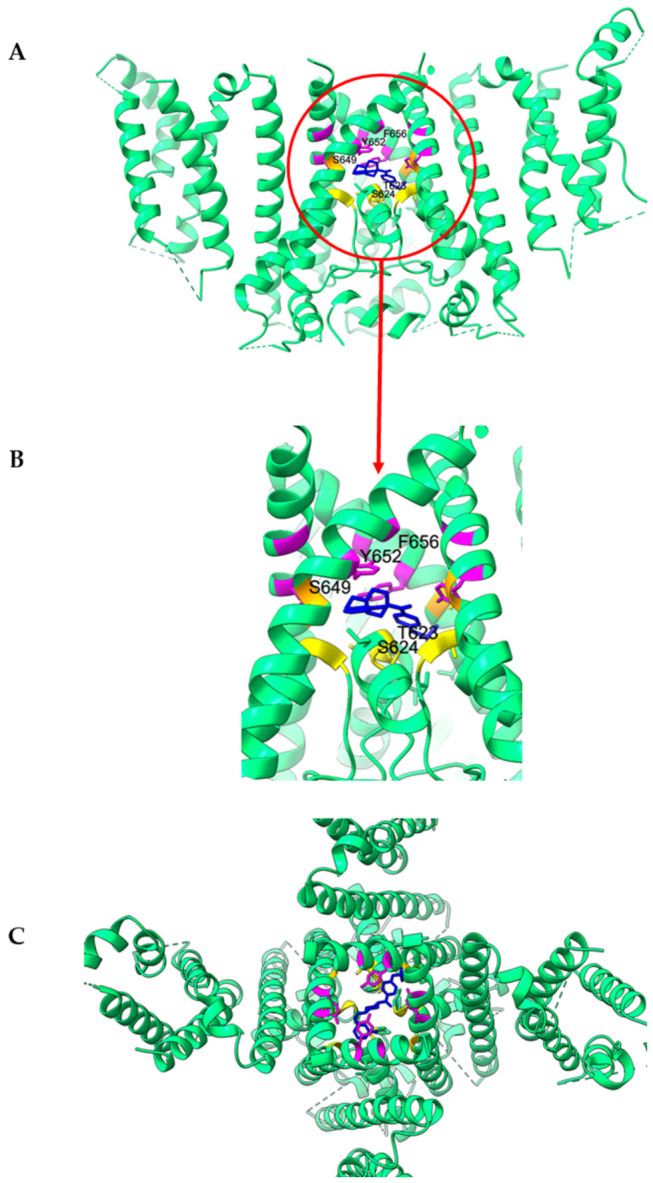
The E-4031-bound form of hERG channel. (**A**): front view of the molecule, (**B**): ligand binding site enlarged, (**C**): top view of the molecule. Structure was colored and labeled by ChimeraX 1.11.1, model used: 8ZYP, “Cryo-EM Structure of E-4031-bound hERG Channel”.

In a recent study, Miyashita et al. [29] utilized Cryo-EM to successfully determine the binding mode of three molecules to the hERG channel. Among the studied ligands, E-4031 contained an N-methanesulfonamide group, whilst the other two (astemizole and pimozide) did not. The common structural element of the three molecules under investigation was a piperidine ring, which (in its protonated state) was found to play a crucial role in blocking the channel. The common structural element of the three molecules under investigation was a piperidine ring, which (in its protonated state) was directly involved in blocking the channel. This is in agreement with several previous pharmacophore models which attribute a critical role to a group containing a positively charged nitrogen atom. The Tyr652 amino acid is involved in hydrophobic or pi-pi interactions with specific groups in all three drugs. (Aromatic and hydrophobic interactions are another similarity with earlier models.) Specifically, in the E-4031 molecule, the methanesulfonamide group formed hydrogen bonds with S624 and S649 in one subunit and T623 in another subunit. The study provides a valuable insight into the structural changes triggered by ligand binding in the hERG channel. However, despite these advances, the complete reconciliation of the cryo-EM results with earlier models remains difficult. A notable unanswered question is the contribution of the Phe656 residue, located in the S6 helix to binding. Mutagenesis and alanine scanning experiments have demonstrated that this amino acid is highly important for binding several channel blockers, including MK-499, dofetilide, ibutilide quinidine and cisapride [18]. Yet it does not show interactions in the cryo-EM structure with any of the three inhibitors. This residue may either have allosteric contributions [29], or alternatively, the channel conformation captured in cryo-EM, although open, does not represent the optimal conformation for high affinity interaction with the molecules whose binding involves Phe565 [30]. Another factor that must be accounted for in in silico models is dissociation kinetics. Some inhibitors such as amiodarone, cisapride, droperidol and haloperidol dissociate relatively rapidly while others can remain trapped even when the channel closes and dissociation takes much longer. This latter group includes inhibitors such as bepridil, domperidone, E-4031 and terfenadine [31]. Therefore, a more complete understanding of the binding mechanisms would require additional cryo-EM structures corresponding to conformations not yet observed. Such new structures could help reconcile the sometimes even partially contradictory findings among the available data. Alternatively detailed molecular modeling, molecular dynamics, or artificial intelligence based protein–ligand structure modelling (such as AlphaFold3 or Boltz-2) could serve as an alternative. The published structures have the potential to serve as a foundation for future in silico studies, which could elucidate the role of the methanesulfonamide group in the channel-blocking properties of these molecules and serve as an even more detailed model for understanding the hERG channel-pharmaceutical binding. This is particularly relevant for drugs whose binding has not yet been studied at the molecular level. In Table 1 the authors collected the most important interacting residues of the hERG channel. Figure 1 as visualization of the drug binding and trapping mechanism of methanesulfonanilide drugs in the channels closed state.

### 1.3. The Importance of Repolarization, the Role of I_Kr_

The ventricular cardiomyocyte action potential (AP) comprises five phases, numbered sequentially from 0 to 4—0th: early, rapid depolarization, 1st: early repolarization, 2nd: plateau phase, 3rd: terminal repolarization, 4th: resting membrane potential. The amplitude and duration of these phases are determined by the delicate interplay of inward and outward ion currents. The rapid component of the delayed rectifier potassium current (I_Kr_) has been identified as a pivotal player in terminal repolarization (phase 3) [32,33,34]. I_Kr_ is due to activation of the pore-forming subunit formed by Kv11.1 (human ether-a-go-go-related gene—hERG) protein [35,36,37]. The alignment of the peak with the commencement of phase 3 is observed in both atrial and ventricular myocytes. Furthermore, the current density of I_Kr_ is proportional to the velocity of the terminal repolarization. Collectively, these data show that these properties significantly influence the duration of the action potential (APD).

AP prolongation caused by impaired repolarization can lead to the development of early afterdepolarizations (EADs) [38], which is a pro-arrhythmic event and might lead to a potentially lethal torsade de pointes (TdP) type of ventricular arrhythmia [39].

AP prolongation is indicated by an increase in the QT interval on the electrocardiogram (ECG). Class III antiarrhythmic agents of Vaughan Williams classification act as inhibitors of I_Kr_ [40], leading to prolongation of the AP and, consequently, the refractory period. This is the mechanism behind the antiarrhythmic action of these compounds. However, for the same reasons, these drugs that increase the APD also have pro-arrhythmic potency under some conditions.

### 1.4. Methanesulfonamide Drugs Targeting the Heart

The most potent class III antiarrhythmic drugs are characterized by the presence of the methanesulfonamide group within their molecular structure. It has been established that the hERG potassium channel of ventricular cardiomyocytes is blocked by trapping inside the inner vestibule of the open channel [18,41]. Consequently, the molecule remains in the closed channel, thereby slowing its dissociation. It can thus be concluded that the molecules in question inhibit I_Kr_, and that the effects of this inhibition are unctionally persistent due to slow dissociation kinetics. The result of this is the potential for the occurrence of arrhythmias. It has been established that, due to this property, drugs which are not primarily antiarrhythmic but contain the methanesulfonamide group are capable of blocking the hERG channel at concentrations that correspond to their therapeutic or “realistic overdose” concentrations. The clinical significance of methanesulfonanilide-mediated hERG blockade extends beyond electrophysiological observations, as excessive I_Kr_ inhibition represents one of the major causes of acquired long QT syndrome and drug-induced Torsades de Pointes. These results indicate that methanesulfonanilide compounds exhibit high affinity and slow dissociation kinetics, which results in the retention of substantial channel occupancy even at declining plasma concentrations. This phenomenon is particularly pronounced in patients with impaired drug elimination.

## 2. Findings

### 2.1. Anti-Arrhythmic Drugs with Methanesulfonamide Group

**Figure 2:**
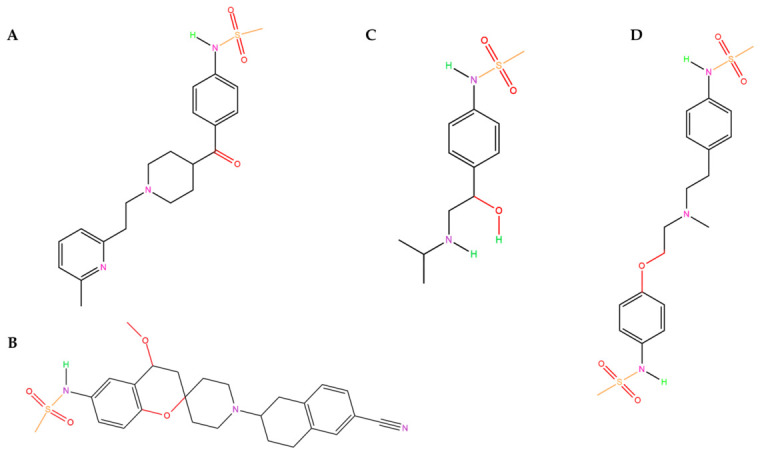
The molecular structure of four methanesulfonanilide drugs synthesized for cardiac use: (**A**): E-4031; (**B**): MK-499; (**C**): d-sotalol; (**D**): dofetilide. Structures were created by ChemDrawPro 12.0 software.

Methanesulfonanilide drugs are archetypal class III antiarrhythmics. Given their classification as cardiac drugs, it is not unexpected that they exert an effect on the heart rhythm. However, given the cardiac AP’s reliance on the delicate equilibrium of ionic currents within myocytes, minor alterations can potentially precipitate cardiac arrhythmias. It is imperative that the actions of cardiac drugs are meticulously documented, encompassing their dosage limits, to ensure their safety and prevent human error from precipitating a catastrophic, potentially lethal arrhythmia. The molecular structure of the four cardiac drugs to be discussed in this review is shown in Figure 2.

#### 2.1.1. Too Potent to Use in Medical Treatment: E-4031 and MK-499

Two hERG-inhibiting compounds have been in the focus of extensive research, yet neither E-4031 nor MK-499 could be released into clinical practice. The utilization of these agents has been limited to a small number of clinical trials, primarily due to their high pro-arrhythmic potency and low reversibility. Consequently, they are currently employed solely in research. The two compounds were introduced in the early 1990s. A significant body of in vitro research has been conducted on these agents, providing substantial evidence for their hERG blocking effect. This effect has been demonstrated in a variety of settings, including single-channel measurements [42] and I_Kr_ inhibition in isolated ventricular cardiomyocytes. The present findings demonstrate that these molecules selectively block the hERG channel in its open state, while maintaining unaltered function in closed channels. It was also determined that MK-499 exhibits a preference for blocking the inactivated state of the channel. Consequently, closure of the activation gate traps the molecule within the channel, thereby increasing the washout period of the effect [43]. E-4031 was also tested in guinea pig atrial and ventricular myocytes in vitro [44]. In this preliminary study, E-4031 was demonstrated to possess a concentration-dependent APD_70_-increasing effect (from the control of 219 ± 6 ms to 239 ± 10, 292 ± 5, and 368 ± 27 ms for 0.03, 0.1, and 0.3 µM E-4031, respectively). 

In the trial of Okada et al., E-4031 was administered to six patients suffering from arrhythmia, with their condition being monitored using a standard 12-lead ECG [45]. The electrocardiogram (ECG) revealed a substantial increase in the corrected QT interval (QTc), from 420 ± 22 ms to 468 ± 41 ms. In four out of six patients treated with E-4031, the QTc interval could not be accurately determined. This was due to the frequent appearance of premature ventricular or supraventricular contractions, which is a more common occurrence than in the case of the other tested drugs.

Conversely, their high affinity (both half-maximal inhibitory concentration (IC_50_) is in the nanomolar range) renders them frequently utilized in hERG research. For instance, MK-499 was utilized in a study by Mitcheson et al. to ascertain the structural target of current inhibition in hERG channel protein [17]. It was determined that missense mutations within the S6 domain of the channel (G648A, Y652A, and F656A) result in the protein exhibiting near-complete resistance to MK-499. E-4031 exhibits high affinity for hERG, selectively blocking it in isolated ventricular cardiomyocytes, often to the point of pro-arrhythmic EADs [46]. Consequently, numerous research laboratories regard the E-4031-sensitive current as control I_Kr_ in isolated ventricular myocytes [32].

#### 2.1.2. The Old and Reliable: D-Sotalol

D-sotalol is considered to be one of the earliest class III antiarrhythmic drugs to be used in clinical practice. The levorotatory isomer, l-sotalol, was first introduced in 1960 as a beta blocker, due to its ability to bind non-selectively to β1 and β2 adrenergic receptors. Nevertheless, subsequent to its debut, research demonstrated that the dextrorotatory isomer, d-sotalol, exerts an antiarrhythmic effect. The antiarrhythmic effect of the drug was studied in two different contexts: firstly, in post-ischemic canine hearts [47] and secondly, in patients suffering from arrhythmia [45,48]. In all studies, sotalol was found to have a significant effect on the occurrence of arrhythmias, with a reduction in incidence observed in all cases. In the study conducted by Anderson et al. [2], the drug was also found to reduce heart rate, thus demonstrating both β-blocking and class III antiarrhythmic actions. Okada et al. [45] demonstrated significant QTc prolongation in the d-sotalol case as well (from 405 ± 35 to 458 ± 29 ms).

In order to further research on the capabilities of d-sotalol as a class III antiarrhythmic agent, Varró et al. [49] also studied it on the AP of isolated canine ventricular Purkinje fibers. The primary outcomes of this study were the substantial prolongation of APD at 50% (APD_50_) and 90% (APD_90_) repolarization (APD_50_: from 176 ± 9 to 206 ± 7 ms, APD_90_: from 249 ± 11 to 326 ± 10 ms).

Subsequent to these endeavors, a relatively recent study by Orvos et al. [50] compared multiple class III antiarrhythmics with manual and automated patch-clamp techniques. That study demonstrated that d-sotalol exerts its effects by selectively inhibiting hERG channels when expressed in immortalized human embryonic kidney (HEK) cells. This inhibition leads to a significant decrease in the average current density of I_Kr_ in isolated rabbit ventricular myocytes, as reported in [50]. The most intriguing discovery in this instance was that its IC_50_ value exhibited variability under differing conditions. During automated patch-clamp experiments conducted at room temperature, the IC_50_ was determined to be 774 ± 9 µM, whereas at 37 °C, it was found to be 343 ± 25 µM. The IC_50_ was determined to be 78 ± 5 µM in HEK cells and 52 ± 9 µM in isolated left ventricular myocytes, both at 37 °C, when performing manual patch-clamp experiments. In the aforementioned study, the effects of d-sotalol on the action potential of rabbit and human ventricular myocytes were measured. In both cases, d-sotalol significantly increased APD_90_. This increase was observed in both rabbit myocytes (56 ± 5% increase from 157 ± 11 to 244 ± 16 ms) and human cells (28 ± 2% increase from 302 ± 20 to 387 ± 28 ms). However, no EADs or delayed afterdepolarizations (DADs) were observed.

#### 2.1.3. New and Stable: Dofetilide

Dofetilide is a methanesulfonamide-based class III antiarrhythmic agent that is currently among the most widely used [51]. A substantial body of research has been conducted on the subject. The therapeutic concentration of the substance under investigation falls within the range of 2 to 7 nM. 

One of the initial studies to assess the efficacy of dofetilide, previously designated as UK-68,798, involved experiments on guinea pig and canine ventricular muscle, as well as canine Purkinje fibers. This investigation was conducted by Gwilt et al. [52], where a significant increase was observed in APD_50_, APD_90_ and effective refractory period on the canine heart preparation. Furthermore, the substance under investigation was found to inhibit both I_K1_ and I_Kr_ of isolated guinea pig ventricular myocytes.

Subsequently, a study was conducted by Kiehn et al. [53] in hERG-expressing Xenopus oocytes utilizing single-channel patch measurements. Although the parameters of the single-channel current remained unaltered from the control conditions, single-channel activity was significantly reduced in dofetilide, indicating a clear effect on the isolated current generated by the hERG channel.

In addition to the aforementioned models of muscle and expressed channels, Okada et al. also conducted a study in which they examined the effects of dofetilide in patients suffering from arrhythmias [45]. Dofetilide was administered to seven patients, with the patients undergoing standard 12-lead ECG monitoring. The QTc interval exhibited a significant increase from 396 ± 15 ms to 457 ± 30 ms. 

In 2001, Weerapura et al. [54] conducted a study in which they examined the hERG channel in the context of dofetilide. However, their primary focus was on the gating-state dependency of the drug’s action. The investigation revealed that dofetilide shares structural similarities with MK-499 and E-4031, requiring the opening of the channel for its therapeutic effects to be manifested. It was also determined that other factors, including membrane voltage and the number of open channels, do not exert an effect on hERG current (IhERG) blocking action. Furthermore, it was established that inactivation mutant hERG channels are less susceptible to the block by dofetilide [54].

Although the literature on dofetilide hERG blocking action and its gating state dependency is extensive, the molecules’ dissociation kinetics have received comparatively less attention. In 2003, Lee et al. [55] conducted a study in which they examined the onset and recovery kinetics of dofetilide on the AP of guinea pig papillary muscles and on the I_hERG_ expressed in Xenopus oocytes. The results of this study indicated that the onset time of 0.1 µM dofetilide effect was 21 ± 2 s, which resulted in an 83 ± 6 millisecond increase in the APD_90_ value. The results demonstrated a 28.0 ± 0.5% recovery during the rest period. This occurred after a period of three seconds without pacing. However, no further recovery was observed. The gradual and incomplete recovery was sustained during the washout period. The authors conducted a study in which they examined the effects of dofetilide on hERG channels that had been expressed in Xenopus oocytes. IhERG exhibited no indications of recovery at any of the applied voltages. The authors of the study interpret this as the result of dofetilide “trapping” inside the closed hERG channel.

In their article [50], Orvos et al. also conducted a study on the effects of dofetilide. A relatively consistent IC_50_ value was identified through the measurement protocols. For automated patch recording at room temperature, the value was found to be 8 ± 0.2 nM. For automated patch recording at 37 °C, the value was determined to be 7 ± 0.2 nM. For manual I_Kr_ recording from isolated myocytes at 37 °C, the value was recorded as 13 ± 3 nM. Dofetilide was observed to have a significant effect on the action potential duration (APD) of isolated cardiomyocytes in rabbits. The APD_90_ increased from 191 ± 14 ms to 286 ± 40 ms, representing a 47 ± 13% increase. In human cardiomyocytes, APD_90_ exhibited an increase from 239 ± 10 to 288 ± 14 ms, representing a 20.4 ± 4.5% rise.

### 2.2. Non-Cardiac Drugs with Pro-Arrhythmic Side Effects

**Figure 3:**
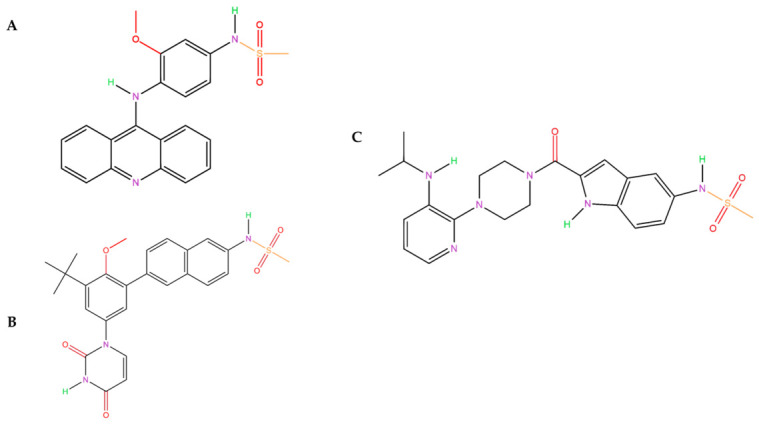
The molecular structure of three methanesulfonamide containing drugs that have hERG blocking effect despite their non-cardiac purpose: (**A**): amsacrine; (**B**): dasabuvir; (**C**): delavirdine. Structures were created by ChemDrawPro 12.0 software.

As previously mentioned, in addition to class III antiarrhythmics, a significant number of other medications have methanesulfonamide in their molecular structure. Rosuvastatin, a pharmaceutical agent employed in the treatment of dyslipidemia, serves as a pertinent example in this regard. The potential for I_Kr_ blocking is evident through channel activity and channel protein expression inhibition [56,57]. As with rosuvastatin, other methanesulfonamide-containing pharmaceuticals have the capacity to exert an effect on the cardiac I_Kr_. The chemical structure of the non-cardiac methanesulfonamide-containing drugs to be discussed subsequently is illustrated in Figure 3.

#### 2.2.1. The Anti-Neoplastic Drug That Blocks hERG: Amsacrine

Amsacrine, first introduced in 1984, functions as a topoisomerase II inhibitor and is used as part of the induction regimen for acute myelogenous leukemia. The substance has been demonstrated to exhibit therapeutic properties within a concentration range of 3 to 18 μM. Cardiac side effects were identified, including QT interval prolongation and arrhythmia [58,59,60,61], yet only a limited number of studies have been conducted thus far regarding its electrophysiological effects [62]. As these are generally linked to I_Kr_ blockade, Thomas et al. sought to ascertain the electrophysiological basis of these adverse effects [63]. In order to achieve this objective, the substance was applied to Xenopus oocytes and HEK cells, which expressed hERG channels. In the experiment conducted on Xenopus oocytes, the agent was tested at concentrations of 1, 10 and 100 µM. The latter inhibited the tail current almost completely, while 10 μM of amsacrine blocked the tail current of the I_hERG_ by over 80%. In this experiment, the authors found that the IC_50_ value of the amsacrine in this model was 2 ± 0.1 μM. Furthermore, it was determined that the substance in question exerts its effect by obstructing the open channel and prolonging the duration of inactivation. This phenomenon is analogous to that observed in other pharmaceutical agents comprising a methanesulfonamide group within their structural composition. Subsequent experiments were conducted on HEK cells to ascertain whether amsacrine could also block hERG in human cells. A notable observation pertaining to the relative tail current measurements was the determination of the IC_50_ value of amsacrine to be 209 ± 6 nM in this second model. However, this discrepancy was not addressed by the authors [63].

#### 2.2.2. The Anti-Hepatitis Agent: Dasabuvir

Dasabuvir is a non-nucleoside hepatitis C virus (HCV) polymerase inhibitor introduced in 2014. The therapeutic concentration of the substance under investigation falls within the range of 0.2 to 4 μM. A study has identified cardiac side effects, including bradycardia, cardiac arrest and chest pain, associated with the administration of dasabuvir-containing anti-HCV medication [64,65,66]. Notwithstanding the existence of the aforementioned evidence, it is notable that no studies of an electrophysiological nature have been conducted with dasabuvir. 

Given the prior experimental experience with E-4031 and dofetilide, there was keen interest in the testing of dasabuvir as well [67]. Firstly, 1 μM dasabuvir was added to the perfusion solution of isolated canine ventricular cardiomyocytes, as APs were being recorded. This modest concentration elicited a substantial increase in APD_90_, escalating from 258 ± 15 to 277 ± 15 ms (representing an 8 ± 3% rise). This observation points to a plausible I_Kr_ block. Furthermore, it was observed that the velocity of phases 0 and 1 of the AP was significantly reduced, indicating that dasabuvir may also exert an influence on ion currents other than I_Kr_. Subsequently the authors assessed dasabuvir at elevated concentrations, ranging from 1 to 30 μM. In the course of the study, it was demonstrated that dasabuvir caused a consecutive increase in APD_90_ (resulting in an approximately 7% (1 μM), 26% (3 μM), 43% (10 μM) and 53% (30 μM) increase). Concurrently, it was observed that dasabuvir decreased the velocity of the terminal repolarization (from −1.8 ± 0.1 to −1.7 ± 0.1, −1.7 ± 0.1, −1.5 ± 0.1, and −1.4 ± 0.1 V/s in 1, 3, 10 and 30 μM, respectively). It was also determined that, at a concentration of 3 µM, the likelihood of EAD occurrence, a discernible pro-arrhythmic factor, was elevated. In order to facilitate the visualization of the dasabuvir-sensitive current beneath the ventricular action potential (AP), a series of AP-clamp measurements were conducted. These measurements revealed the presence of multiple components within the dasabuvir-sensitive current. The first component is of particular interest, as it elucidates the alterations that occur in the early phases of the AP. A subsequent component exhibits maximal density, which corresponds to the V-max value (the maximal rate of phase 3 repolarization) of the AP of the recorded cell. This finding suggests that the component in question can be due to I_Kr_ blockade. Subsequent to the washout of dasabuvir, the remaining current exhibited a configuration analogous to the I_Kr_ of canine cardiomyocytes. This finding has the potential to provide further support for the prevailing theory on the trapping mechanism of the methanesulfonamide drugs. In order to ensure the validity of the results, conventional voltage-clamp experiments were conducted on hERG channels, which were expressed in HEK cells. The antiviral agent dasabuvir, when administered at a concentration of 30 µM, resulted in the near-complete inhibition of the current. However, subsequent to the removal of the agent, the effect was found to be almost completely irreversible. In order to verify the concentration-dependent inhibition of the canine AP, 1, 3, 10 and 30 μM of dasabuvir were applied to HEK cells, where the remaining current fractions were 0.93 ± 0.04, 0.63 ± 0.10, 0.26 ± 0.04, and 0.15 ± 0.02, respectively. Consequently, the IC_50_ value was determined to be 3.2 μM [67].

#### 2.2.3. Fighting HIV: Delavirdine

Following the findings obtained with dasabuvir, the focus was shifted to delavirdine [68], a non-nucleoside reverse transcriptase inhibitor that was first approved in 1997. The therapeutic concentration of delavirdine was found to range from 7 to 33 μM. Initially, conventional voltage-clamp measurements were conducted on HEK cells that stably expressed hERG channels. A total of 30 μM of delavirdine almost fully blocked the hERG current once more, but this effect was fully reversible after washout. Following the administration of delavirdine at a series of concentrations, a concentration-response curve was constructed, yielding an IC_50_ value of 11 ± 1 μM. Delavirdine, at a concentration of 10 μM, was observed to induce a significant prolongation in the action potential duration (APD_90_) of isolated canine left ventricular cardiomyocytes. The APD_90_ increased from 204 ± 10 ms to 226 ± 13 ms, representing an approximate increase of 11%. This effect was found to be reversible, with the APD_90_ returning to 21 1 ± 12 ms following the removal of the drug. Furthermore, the maximal rates of the 0th, 1st and 3rd phases of the AP were also reduced by the substance (−25%, −34% and −28% respectively). Subsequent to this, the 1–3–10–30 μM sequence was utilized in conjunction with delavirdine. The APD_90_ increased in proportion to the following extents: 8%, 15%, 23% and 37%, respectively. Delavirdine has been demonstrated to reduce the maximal rate of repolarization of the first and third phases of the AP, in a concentration-dependent manner. Delavirdine-sensitive currents exhibited a more distinct configuration in comparison to dasabuvir-sensitive currents as evidenced by AP-clamp measurements. The current is composed of two distinct components, which are distinctly separated. The former is characterized by its similarity to the I_to1_ current of the cardiomyocytes, while the latter is analogous to the I_Kr_. The early component reaches its maximum peak value approximately five minutes after the commencement of drug perfusion; however, the late component requires a further five minutes to reach its maximum [68].

## 3. Conclusions

The present review highlights that methanesulfonamide-containing compounds represent one of the best-characterized classes of high-affinity hERG potassium channel blockers. As evidenced by the examples of methanesulfonanilide drugs presented, the inhibition of cardiac I_Kr_ through hERG channel block appears to be a well-founded phenomenon. Although hERG inhibition is not exclusive to methanesulfonamide-containing molecules, accumulating electrophysiological, structural and also in silico evidence suggests that this functional group contributes significantly to the characteristic binding behavior of classical methanesulfonanilide compounds such as dofetilide, E-4031 and MK-499. The mechanism of action in question has been extensively studied, with a significant number of articles reporting that the channels are exclusively blocked in their open state. The experimental studies demonstrated that these compounds interact with residues located within the inner vestibule of the hERG pore, particularly Y652 and F656. Structural analyses and docking studies identified hydrogen bond interactions involving residues T623, S624 and S649 also located within the inner vestibule. As demonstrated by the two aforementioned cryo-EM studies, the methanesulfonamide binding sites exhibit a high degree of consistency amongst the molecules [28,29]. Moreover, observations have been made pertaining to the formation of hydrogen bonds between the methanesulfonamide and its binding sites. It has been demonstrated that the molecules exhibit a reduced propensity to bind within the hydrophobic pocket. While it is evident that other drugs, devoid of the aforementioned structural element, are capable of inhibiting hERG channel activity, these findings may serve to underpin the high affinity of methanesulfonamide drugs for the channel and their capacity to trap in them. It is evident that these interactions collectively contribute to the facilitation of stable pore occupancy, high-affinity inhibition, and the unusually slow dissociation kinetics that are characteristic of methanesulfonanilide blockers. The capacity of a number of compounds to become entrapped within the closed channel state further differentiates this pharmacological class from numerous other hERG inhibitors [41,45,50]. Consequently, these substances can function as effective agents in the treatment of various cardiac tachyarrhythmias, which are classified as class III antiarrhythmic medications. However, it can also be stated that a significant proportion of these agents exhibit a high degree of potency in terms of their binding affinity for the hERG channel, as evidenced by the examples of E-4031 [46] and MK-499 [17]. From a physiological perspective, excessive hERG blockade suppresses the rapid delayed rectifier potassium current (I_Kr_), prolongs ventricular repolarization and increases the risk of QT interval prolongation and development of Torsades de Pointes. It is important to note that the high affinity and prolonged channel occupancy of methanesulfonanilide compounds may become particularly relevant under conditions associated with impaired drug elimination, including renal or hepatic dysfunction. In such cases, accumulation can further increase proarrhythmic risk. It has been well documented that non-cardiac pharmaceuticals containing the methanesulfonamide group are capable of exerting a toxic effect on the heart. In such cases, the pharmaceutical agents primarily employed are anti-viral or anti-neoplastic drugs, including delavirdine [68] and amsacrine [63].

These drugs have the capacity to reach and inhibit the cardiac hERG channels, thereby inducing a wide variety of arrhythmic events. The properties of the compounds mentioned in the results section are summarized in Table 2. In addition to their toxicological significance, methanesulfonanilide compounds offer valuable mechanistic insight into the structural determinants of hERG channel pharmacology, as demonstrated by cryo-EM molecular docking and computational modelling studies.

**Figure 4:**
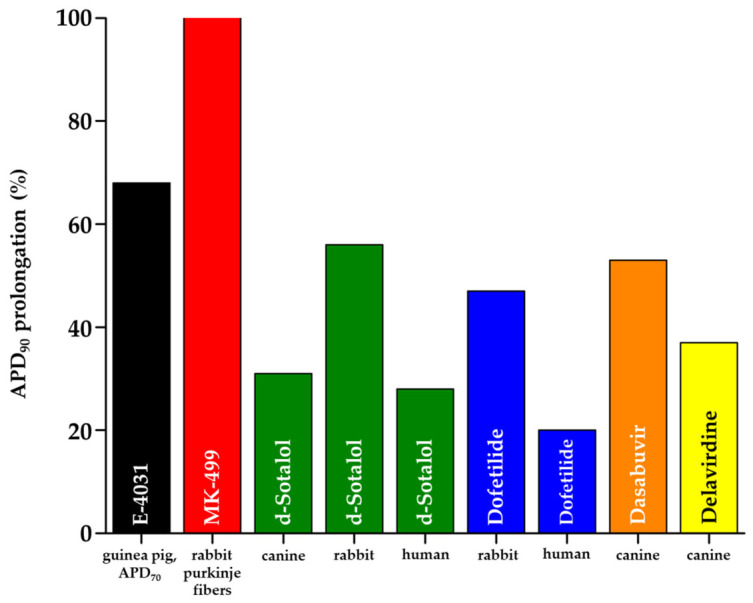
The strongest APD_90_ changing capability (% of control) of substances where data were avaible.

Extant evidence indicates that the methanesulfonamide moiety acts as an affinity-enhancing structural contributor rather than a universal hERG pharmacophore. Rather, it should be considered an important structural contributor to the characteristic high-affinity and state-dependent blockade exhibited by several clinically relevant and experimentally important hERG inhibitors. The IC_50_ values, dosage and APD_90_ prolongating ability of the discussed compounds are summarized in Table 2, and the latter one is visualized on Figure 4.

## 4. Future Developments

The future of methanesulfonanilide drugs appears to be uncertain. The primary objective is to eradicate or, at the very least, substantially minimize the pro-arrhythmic side effects of both cardiac and non-cardiac medications. In the domain of cardiac pharmacology, research initiatives are predominantly directed towards the reduction in hERG affinity. The principal aim of this approach is to reduce the likelihood of drug trapping, whilst preserving the anti-arrhythmic efficacy of the medication. This strategy is intended to ensure the continued efficacy of the drug, thereby preventing the occurrence of pro-arrhythmia, which can lead to more predictable medication outcomes. Conversely, non-cardiac pharmaceuticals are designed to impede hERG binding, thereby mitigating the occurrence of cardiac adverse effects. In both cases, researchers typically undertake the removal of methanesulfonamide from the molecule in order to achieve their objective. In 2023, Helliwell et al. [76] conducted a study in which they tested a methanesulfonamide-free analogue of E-4031, designated E-4031-17. The results obtained revealed the presence of wild-type and several mutant hERG channels in HEK 293 cells, and conventional voltage-clamp measurements were then conducted on these cells. It was determined that the elimination of the methanesulfonamide group did not result in any alteration to the hERG-blocking effect of the substance. However, it was found to be more readily dissociated from the channel during washout periods. Nevertheless, through the utilization of multiple applications, the block was observed to be capable of accumulation. This demonstrated the importance of further exploration of these altered molecules, as it will result in the identification of more use-dependent agents, which in turn will facilitate more straightforward medication planning. Conversely, Carvalho et al. (2013) [77] utilized 72 aromatic analogues of dofetilide, comprising a central pyridine moiety and devoid of methanesulfonamide, to identify some with diminished hERG binding affinity, thereby reducing the likelihood of cardiac adverse effects. The researchers employed radioligand binding and patch-clamp assays in their experimental design. It has been demonstrated that several of these analogues possess IC_50_ values that are comparable to those of the most potent hERG blockers. Nevertheless, permanently positively charged pyridines have been observed to demonstrate reduced hERG affinity. However, it should be noted that this method has not been deemed a reliable one. It was demonstrated that an increased rigidity, attributable to triple bonds, was a more reliable modification, thereby mitigating activity on hERG.

These studies demonstrated that the methanesulfonamide functional group is an integral component of the hERG channel blocking effect, capable of eliciting both positive and negative effects.

In addition to the conventional research methods demonstrated in previous studies, the rapidly expanding databases of protein structures suggest that machine learning models could be considered a novel approach to researching drug–protein interactions. The domain of machine learning has witnessed recent advancements in the field of drug–drug interactions, as reviewed by Lu et al. [78]. The review categorizes machine learning (ML)-based drug–drug interaction (DDI) prediction methods into several primary types, some of which have the potential to be utilized in the research of drug–protein interactions as well, such as learning paradigms. However, it should be noted that there are certain disadvantages associated with this approach. These include the potential ambiguity of the internal decision-making processes employed by these models and the challenges associated with clinical translation. Nevertheless, the integration of large language models and generative models has the potential to position machine learning as a potent instrument in the research of drug–protein interactions, as Liu et al. [79] researched various hERG interactions, first with six individual QSAR models then with three ensemble models as well. All models achieved high performance with Matthews correlation coefficient (MCC) values between 0.682 and 0.730, exceeding the best previously reported MCC of 0.689 on the same dataset. Also, external validation on EV-1 (biased toward blockers) demonstrated strong extrapolation ability, with MCC values from 0.520 to 0.715, significantly better than prior models (0 to 0.599). This study successfully developed robust machine learning and deep learning QSAR models using Mold2 descriptors to predict hERG channel blockade with improved accuracy and generalization compared to existing models. The ensemble approaches, particularly including all six models, provided enhanced predictive performance in cross-validation. These models offer valuable computational tools to support pharmaceutical industry by reducing the risk of cardiac adverse effects of new drug candidates.

## Figures and Tables

**Table 1:** Reported interacting residues involved in methanesulfonanilide binding to the hERG potassium channel.

Interacting Residue	Interaction Type	Tested Compounds	Reference
T623	H-bond, aromatic residue	E-4031, Dofetilide	Miyashita et al. [29], Kamiya et al. [18]
S624	H-bond, aromatic residue	E-4031, Dofetilide	Miyashita et al. [29], Kamiya et al. [18]
S649	H-bond, aromatic residue	E-4031	Miyashita et al. [29]
Y652	H-bond, aromatic residue	E-4031, Dofetilide, MK-499	Miyashita et al. [29], Kamiya et al. [18], Mitcheson et al. [17]
F656	H-bond, aromatic residue	E-4031, Dofetilide, MK-499	Miyashita et al. [29], Kamiya et al. [18], Mitcheson et al. [17]

**Table 2:** The drugs discussed in the review, their IC_50_ value on hERG channels, therapeutic dosage (if applicable) and their APD_90_ prolonging effect.

Drug Name	IC_50_	Dosage	APD_90_ Prolongation	References
E-4031	7.7 nM	no data	9–68% (guinea-pig, APD_70_)	Zhou et al. [69], Wettwer et al. [44]
MK-499	21.4 nM	no data	~100% (rabbit purkinje fibers)	Coi et al. [70], Cordeiro et al. [71]
d-Sotalol	51.6–773.7 µM	80–640 mg/day	31% (canine), 56% (rabbit), 28% (human)	drugs.com [72], Varró et al. [49], Orvos et al. [50]
Dofetilide	8.4–13.0 nM	2 × 125–500 µg/day	47% (rabbit), 20% (human)	medscape.com [51], Orvos et al. [50]
Amsacrine	2.0 nM, 209.4 nM	150–340 mg/day	no data	Arlin et al. [73], Thomas et al. [63]
Dasabuvir	3.2 µM	2 × 250 mg/day	7–53% (canine)	drugs.com [74], Kovács et al. [67]
Delavirdine	11.0 µM	3 × 400 mg/day	8–37% (canine)	drugs.com [75], Óvári et al. [68]

## Data Availability

No new data were created or analyzed in this study. Data sharing is not applicable to this article.

## Abbreviations

The following abbreviations are used in this manuscript:

AP	Action Potential
APD	Action Potential Duration
APD_50_	Action Potential Duration at 50% repolarization
APD_70_	Action Potential Duration at 70% repolarization
APD_90_	Action Potential Duration at 90% repolarization
COX-2	Cyclooxygenase-2
Cryo-EM	Cryo-electron microscopy
DAD	Delayed Afterdepolarizations
DDI	Drug–Drug Interaction
EAD	Early Afterdepolarizations
ECG	Electrocardiogram
HCV	Hepatitis C Virus
HEK	Human Embryonic Kidney (cells)
hERG	Human Ether-a-go-go-Related Gene
IC_50_	Half Maximal Inhibitory Concentration
I_Kr_	Rapid Component of the Delayed Rectifier Potassium Current
I_to1_	Transient Outward Potassium Current 1
Kv11.1	Potassium Channel Subunit Forming hERG Channel
MCC	Matthews correlation coefficient
ML	Machine Learning
QSAR	Quantitative Structure–Activity Relationship
QTc	Corrected QT interval
TdP	Torsade de Pointes
